# Free cyanide and thiocyanate biodegradation by *Pseudomonas aeruginosa* STK 03 capable of heterotrophic nitrification under alkaline conditions

**DOI:** 10.1007/s13205-015-0317-2

**Published:** 2015-12-31

**Authors:** Lukhanyo Mekuto, Seteno Karabo Obed Ntwampe, Margaret Kena, Mhlangabezi Tolbert Golela, Olusola Solomon Amodu

**Affiliations:** Bioresource Engineering Research Group (BioERG), Department of Biotechnology, Cape Peninsula University of Technology, PO Box 652, Cape Town, 8000 South Africa

**Keywords:** Biodegradation, Cyanide, Heterotrophic nitrification, *Pseudomonas aeruginosa* STK 03, Thiocyanate

## Abstract

An alkali-tolerant bacterium, *Pseudomonas aeruginosa* STK 03 (accession number KR011154), isolated from an oil spill site, was evaluated for the biodegradation of free cyanide and thiocyanate under alkaline conditions. The organism had a free cyanide degradation efficiency of 80 and 32 % from an initial concentration of 250 and 450 mg CN^−^/L, respectively. Additionally, the organism was able to degrade thiocyanate, achieving a degradation efficiency of 78 and 98 % from non- and free cyanide spiked cultures, respectively. The organism was capable of heterotrophic nitrification but was unable to denitrify aerobically. The organism was unable to degrade free cyanide in the absence of a carbon source, but it was able to degrade thiocyanate heterotrophically, achieving a degradation efficiency of 79 % from an initial concentration of 250 mg SCN^−^/L. Further increases in thiocyanate degradation efficiency were only observed when the cultures were spiked with free cyanide (50 mg CN^−^/L), achieving a degradation efficiency of 98 % from an initial concentration of 250 mg SCN^−^/L. This is the first study to report free cyanide and thiocyanate degradation by *Pseudomonas aeruginosa*. The higher free cyanide and thiocyanate tolerance of the isolate STK 03, which surpasses the stipulated tolerance threshold of 200 mg CN^−^/L for most organisms, could be valuable in microbial consortia for the degradation of cyanides in an industrial setting.

## Introduction

Natural and anthropogenic activities contribute to cyanide and thiocyanate (SCN^−^) contamination in the environment. However, a significant source of cyanide contamination is through anthropogenic activities such as the cyanidation process, which is used in the mining industry to extract precious metals such as gold and silver from refractory sulphidic ores (Gould et al. 2012). Since free cyanide (CN^−^) is a highly reactive chemical, it also reacts with a number of metals that are present within the ore forming metal-complexed cyanides which are categorised as: weak acid dissociable and strong acid dissociable cyanides (Mudder et al. 2001). Additionally, cyanide reacts with sulphur species present within the ore, thus forming significant concentrations of thiocyanate which can be up to 3000 mg SCN^−^/L (Stott et al. 2001; van Hille et al. 2015; van Zyl et al. 2015). These compounds contribute significantly to environmental deterioration as many living organisms are susceptible to cyanide compounds. The presence of these compounds has been associated with wildlife mortalities (Donato et al. 2007) and wastewater treatment plant failures as a result of the CN/SCN susceptibility of the organisms which are normally employed in such systems (Kim et al. 2008 a, b; Han et al. 2014). Cyanide is mostly removed in industrial effluents by, amongst others, alkaline chlorination or hydrogen peroxide or ozonation. However, these methods have proven to be environmentally deteriorative as they produce end-products which are hazardous to the environment (Botz et al. 2005; Mudder and Botz 2004). More attention has been shifted to the biotechnological approach for the degradation of cyanide and thiocyanate as it is cost effective, environmentally benign and does not produce end-products which are hazardous to the environment (Akcil and Mudder 2003; Akcil 2003; Patil and Paknikar 1999). The existence of CN/SCN resistant, tolerant and degrading bacterial and fungal organisms has contributed significantly to the development of an effective degradation process, through understanding the microbiological contributions of individual organisms such that accurate predictive models can be developed (Stott et al. 2001). Individually, each specie in a consortia possess specific enzymes and is able to use either hydrolytic, substitution/transfer, reductive and oxidative pathways for the degradation of cyanides (Ebbs 2004).

A number of studies have been reported on bacterial decomposition of cyanide and thiocyanate, and organisms such as *Bacillus pumilus*, *Klebsiella oxytoca*, *Burkholderia cepacia*, *Rhodococcus* ssp, *Thiobacillus* ssp, *Halomonas* ssp and many other organisms have the potential to degrade cyanide and thiocyanate (Adjei and Ohta 2000; Kao et al. 2003; Meyers et al. 1991; Stott et al. 2001; Maniyam et al. 2011). Organisms belonging to the Pseudomonadaceae family have also been reported to degrade cyanide, thiocyanate and metal-complexed cyanides. Organisms such as *Pseudomonas stutzeri*, *Pseudomonas putida*, *Pseudomonas pseudoalcaligenes*, *Pseudomonas flourescens* have been observed as cyanide and thiocyanate degraders (Grigor’eva et al. 2006, 2008, 2009; Karavaiko et al. 2000, Luque-Almagro et al. 2005). However, free cyanide and thiocyanate degradation by a *Pseudomonas aeruginosa* strain has never been reported. Additionally, the effect of free cyanide on thiocyanate biodegradation by *P. aeruginosa* has never been reported. Hence, the primary aim of this study was to investigate free cyanide and thiocyanate biodegradation ability of an isolate, *Pseudomonas aeruginosa* STK 03.

## Materials and methods

### Microorganism and inoculum preparation

A bacterium that was able to grow on free cyanide and thiocyanate containing media was isolated from a site in Nigeria contaminated with cyano group containing compounds including poly aromatic hydrocarbons. The isolate was denoted as STK 03. The organism was isolated using a culture-based technique. A serial dilution on sterile saline solution was performed on the original sample and plated on nutrient agar plates containing 100 mg CN^−^/L at 30 °C for 48 h. This was done to selectively isolate cyanide-tolerant organisms. Identification of the organism was performed using the 16S rDNA sequencing followed by Polymerase Chain Reaction (PCR) using bacterial universal primers. The DNA was extracted using a ZR Fungal/Bacterial DNA Kit (Zymo Research, California, USA). The presence of the genomic DNA was assessed using a 1 % (w/v) molecular grade agarose gel containing 0.5 μg/mL ethidium bromide (EtBr), using 1X Tris–acetate–ethylenediamine tetraacetic acid (TAE) electrophoresis buffer at 100 V for 1 h. PCR was performed using a GeneAmp PCR 9700 System (Applied Biosystems, USA). Amplification of the target DNA by PCR was performed in a total reaction volume of 10 μL containing 0.5 μL (±50 ng/μL) of the purified genomic DNA, 50 mM of the forward and reverse primers and 5 μL of a 2X KapaTaq Readymix solution (KapaBiosystems, South Africa). Bacterial-specific primers used were the forward 8F primer 5′-AGAGTTTGATCCTGGCTCAG-3′ and reverse primer 1492R 5′-GGTTACCTTGTTACGACTT-3′. The amplification process included an initial denaturing step at 94 °C for 10 min, followed by 36 cycles of 94 °C for 30 s, 55 °C for 30 s and 72 °C for 1 min. The reaction was completed with a final extension period of 7 min at 72 °C followed by cooling and storage at 4 °C. PCR amplicons (10 µL) were electrophoretically analysed on a 1 % (w/v) molecular grade agarose gel that was stained with ethidium bromide, using 1× TAE electrophoresis buffer at 100 V for 1 h, to determine whether the amplification was successful. The PCR amplicons were run on an ABI 3010xl Genetic analyser. The sequences were blasted against the NCBI GenBank database (www.ncbi.nlm.nih.gov) and the sequences were deposited on the NCBI gene bank database. The isolate was allocated an accession number, KR011154.

For both free cyanide and thiocyanate degradation studies, the organism was grown for a period of 48 h in minimal media (MM) that contained (g/L): K_2_HPO_4_ (4.3), KH_2_PO_4_ (3.4), MgCl·6H_2_O (0.4) and whey waste (1.4). The pH of the media was adjusted to an initial pH of 10 for free cyanide studies and 8.5 for thiocyanate studies, with pH not being controlled thereafter. The organism was unable to degrade thiocyanate above a pH of 8.5 (data not shown); hence, the pH was set at 8.5 for SCN^−^ degradation studies. The media did not contain any nitrogen source and a mature culture was used as an inoculum, representing 10 % (v/v) of the total volume used for the biodegradation studies.

### Experimental plan

The organism was inoculated in MM which was supplemented with free cyanide (as KCN) at concentrations of 250 and 450 mg CN^−^/L, and thiocyanate (as KSCN) at 250 mg SCN^−^/L, in a total working volume of 200 mL. The uninoculated bioreactors served as controls. The bioreactors were incubated in an orbital shaker at 180 rpm and 30 °C. Cyanide and thiocyanate studies were run separately. Free cyanide studies were ran in airtight shake flasks fitted with sampling ports while thiocyanate studies were ran in Erlenmeyer flasks. The use of airtight flasks was done to minimise cyanide volatilisation. To demonstrate nitrification and aerobic denitrification, the isolate was inoculated onto 200 mL Erlenmeyer flasks with MM medium, containing an initial concentration of ammonium (as NH_4_Cl) and nitrate (as NaNO_3_) of 300 mg NH_4_^+^/L and 100 mg NO_3_^−^-N/L, respectively. The initial pH was set at a pH of 10 for both the nitrification and denitrification studies. Aliquots (2 mL) were periodically withdrawn from the flasks and analysed for free cyanide, thiocyanate, ammonium, nitrates and sulphates as described in “Analytical methods ”.

### Biological cyanide removal efficiency

A mass balance equation for the determination of the biologically degraded cyanide, taking into account cyanide volatilisation, is shown in Eqs. 1 and 2.

1 $$ {\text{CN}}_{\text{S}}^- - ({{\text{CN}}_{\text{R}}^- + {\text{CN}}_{\text{V}}^- }) = {\text{CN}}_{\text{B}}^- $$

where

2 $$ {\text{CN}}_{\text{V}}^- = (   {\text{CN}}_{\text{Vo}}^- - {\text{CN}}_{\text{Vf}}^- )$$

Biological removal efficiency (BRE) was determined according to Eq. 3

3 $$ {\text{BRE }}(\% ) \, = \, \frac{{{\text{CN}}_{\text{B}}^- }}{{{\text{CN}}_{\text{S}}^- }} \times 100$$

where ${\text{CN}}_{\text{B}}^-$ is the biologically degraded cyanide (mg CN^−^/L), ${\text{CN}}_{\text{S}}^-$ is the initial free cyanide concentration in the media (mg CN^−^/L), ${\text{CN}}_{\text{R}}^-$ is the residual free cyanide measured in the inoculated media (mg CN^−^/L), ${\text{CN}}_{\text{V}}^-$ is the cyanide that volatilised during culture incubation (mg CN^−^/L), ${\text{CN}}_{\text{Vo}}^-$ is the initial cyanide concentration in the control cultures (mg CN^−^/L), and ${\text{CN}}_{\text{Vf  }}^-$ is the final cyanide concentration in the control cultures (mg CN^−^/L).

### Statistical analysis

The experimental error was calculated as the standard error of mean using the standard deviation obtained from the multiple sets of data (*n* = 2), as demonstrated in Eq. 4:

4 $$ {\text{SEM = }}\frac{\text{Standard deviation}}{{\sqrt {\text{number of samples tested}} }}$$

### Analytical methods

Merck ammonium (NH_4_^+^) (00683), cyanide (CN^−^) (09701), nitrate (14773) and sulphate (00617) test kits were used to quantify the concentration of free cyanide, ammonium, and nitrates using a Merck Spectroquant Nova 60 instrument. Briefly, the cyanide test kit works on the reaction of cyanide with chloramine-T and pyridine-barbituric acid. The ammonium test kit works on the Berthelot reaction between ammonium ions, chlorine and phenolic compounds to form indophenol dyes. The nitrate test kit makes use of concentrated sulphuric acid in the presence of a benzoic acid derivative while the sulphate test kit makes use of the reaction between sulphates and barium ions and the sulphates are measured turbidimetrically. Nitrites were determined according to the method of Rider and Mellon (1946). The pH was measured using a Crison Basic20 pH meter which was calibrated daily. The microbial population was quantified using a Jenway 6715 UV/visible spectrophotometer at a wavelength of 600 nm. Thiocyanate was quantified using the ferric method (Hovinen et al. 1999).

## Results and discussion

**Fig. 1:**
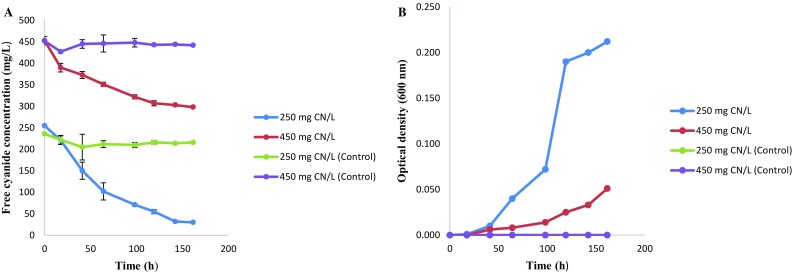
Free cyanide degradation profile at different concentrations and growth profile of *Pseudomonas aeruginosa* STK 03 (**a**) and growth patterns of the organism (**b**). *Error bars* represent deviations

In this study, a free cyanide and thiocyanate tolerant bacterium was isolated and identified as *Pseudomonas aeruginosa* STK 03. Free cyanide biodegradation by *Pseudomonas aeruginosa* STK 03 and growth patterns in MM is shown in Fig. 1a, b, respectively. The organism was able to degrade 250 and 450 mg CN^−^/L, achieving a BRE of 80 and 32 % within 150 h, respectively. Recently, it has been reported that an active aerobic degradation process has a maximum cyanide threshold concentration of 200 mg CN^−^/L (Kuyucak and Akcil 2013). However, in this study, *Pseudomonas aeruginosa* STK 03 was able to degrade free cyanide in cultures containing cyanide concentrations above 200 mg CN^−^/L. Free cyanide degradation was accompanied by growth of the organism, with the initial cyanide having a negative impact on the growth of the organism. The cultures that had low cyanide concentrations showed a shorter lag phase while the cultures with a higher concentration demonstrated a prolonged lag phase. This phenomenon was observed elsewhere (Mekuto et al. 2013), where a *Bacillus* consortia showed varying lag phases with respect to different initial cyanide concentrations, with cultures with the higher concentrations showing a prolonged lag phase. The prolonged lag phase with an increase in free cyanide concentration was a result of cyanide inhibition on microbial growth.

**Fig. 2:**
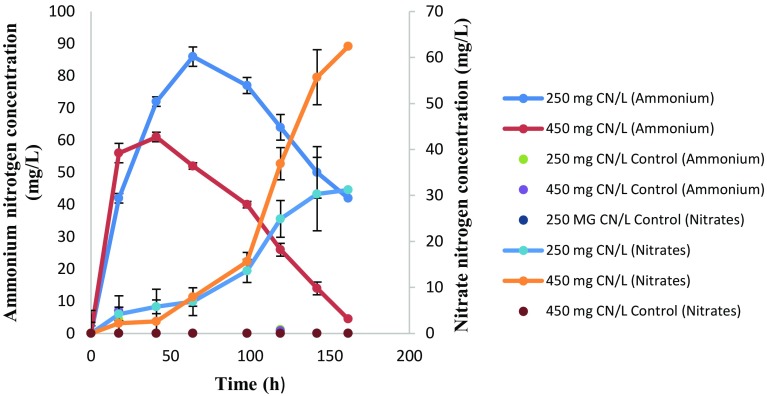
Ammonium nitrogen and nitrate nitrogen profiles as a function of time. *Error bars* represent deviations

**Fig. 3:**
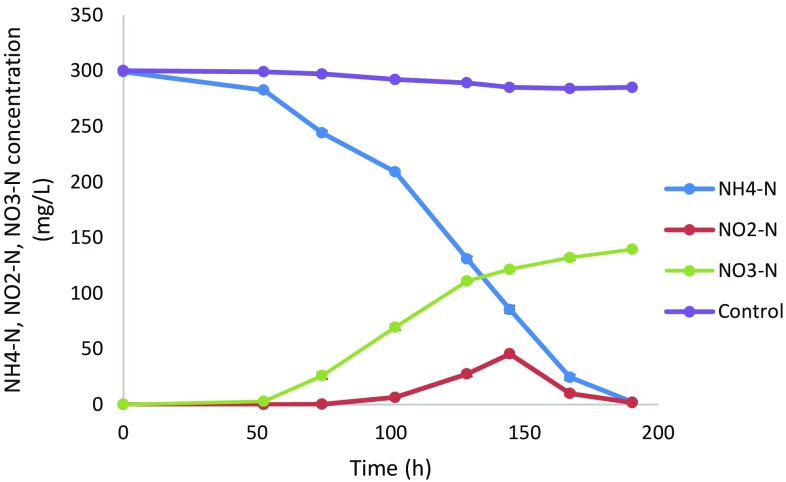
Heterotrophic nitrification profile as a function of time. *Error bars* represent deviations

The degradation of free cyanide resulted in the accumulation of ammonium in the medium, which suggested a possible hydrolytic mechanism of cyanide degradation (Ebbs 2004; Akcil et al. 2003). The maximum ammonium nitrogen concentration from the cultures that had an initial cyanide concentration of 250 and 450 mg CN^−^/L was 86 and 61 mg NH_4_^+^-N/L, respectively. Subsequently, the nitrate nitrogen concentration accumulated in the media, with an observed maximum nitrate nitrogen concentration of 31.2 and 62.4 mg NO_3_^−^-N/L being observed, respectively (see Fig. 2). The ammonium nitrogen concentration decreased after 64 and 41 h from both cultures, with the residual ammonium nitrogen concentration being 42 and 4.5 mg NH_4_^+^-N/L from the cultures that contained an initial cyanide concentration of 250 and 450 mg CN^−^/L, respectively. This showed heterotrophic nitrification capability of *Pseudomonas aeruginosa* STK 03. However, the organism was unable to remove nitrates, thus demonstrating the incapability of the organism to carry out aerobic denitrification. However, *Pseudomonas stutzeri* C3 was found to be able to carry out aerobic denitrification but was unable to carry out heterotrophic nitrification (Ji et al. 2015), while in a separate study *Pseudomonas stutzeri* YZN-001 was able to carry out nitrification and aerobic denitrification (Zhang et al. 2011); a suggestion that isolate STK 03 does not possess denitrification characteristics that are responsible for total nitrogen removal in cyanide-contaminated effluent. To prove heterotrophic nitrification and aerobic denitrification, both ammonium (as NH_4_Cl) and nitrate (as NaNO_3_) were used as nitrogen sources, in separate studies. STK 03 was able to carry out nitrification (see Fig. 3), achieving a nitrification rate of 1.56 mg NH_4_^+^-N L^−1^ h^−1^ with subsequent production and accumulation of nitrates and nitrites while ammonium stripping was determined to amount to 15 %. Both nitrates and nitrites increased during the nitrification stage; however, the concentration of nitrites decreased to 1.75 mg NO_2_^−^-N/L after 150 h, with the accumulation of nitrates being observed (Table 1).

**Table 1:** Aerobic denitrification by *Pseudomonas aeruginosa* STK 03

Time (h)	NO_3_^−^-N (mg/L)	NO_2_^−^N (mg/L)
0	100	0
24	97	0
72	96	0
96	96	0

**Fig. 4:**
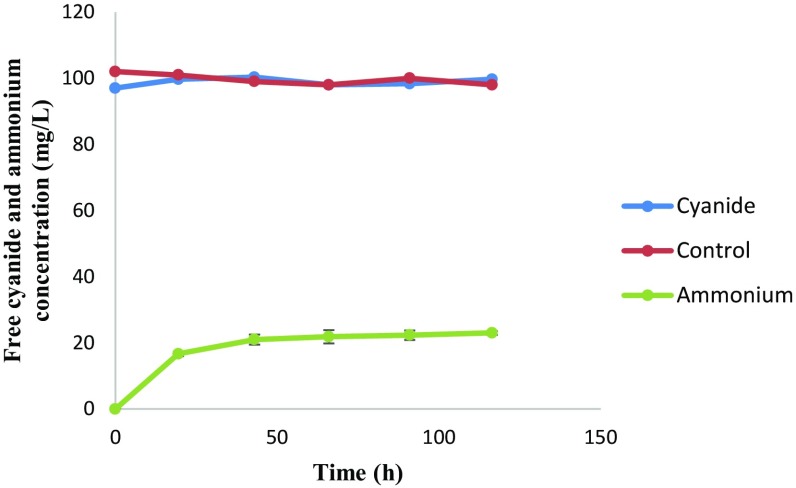
Autotrophic degradation of free cyanide and ammonium nitrogen formation profile. *Error bars* represent deviations

*Pseudomonas aeruginosa* STK 03 was unable to degrade cyanide without the presence of a carbon source, i.e. whey waste (Fig. 4). In the presence of a carbon source, there was a logarithmic increase of ammonium nitrogen from 0 to 40 h and, thereafter, the ammonium concentration reached a plateau. The detection of ammonium nitrogen in the media was due to cell death or disruption and subsequent release of ammonium-related compounds due to cyanide toxicity. This meant that STK 03 was unable to use cyanide as a carbon and nitrogen source and, therefore, an external carbon source was necessary to meet the carbon source requirements of the organism.

**Fig. 5:**
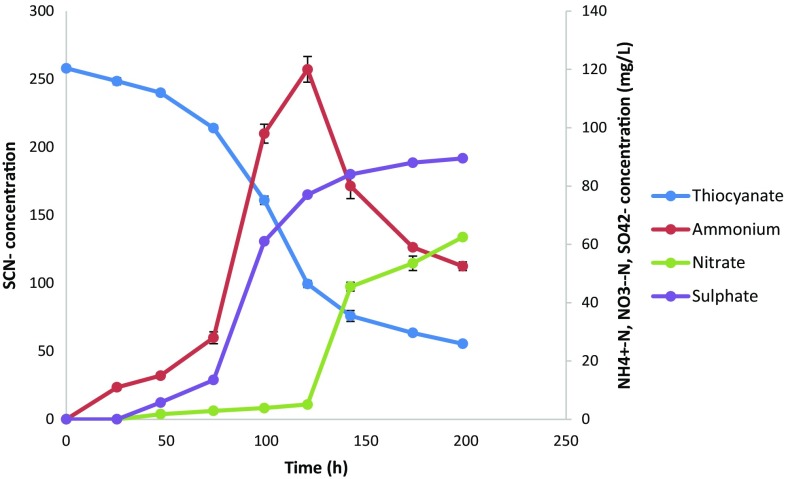
Thiocyanate degradation profile and formation of degradation products without the presence of free cyanide. *Error bars* represent deviations

The capability of the isolate to degrade thiocyanate was evaluated in batch cultures and the organism was able to degrade 250 mg SCN^−^/L to 55.5 mg SCN^−^/L over a period of 200 h (Fig. 5). This is equivalent to a degradation efficiency of 78 %. Thiocyanate degradation resulted in the accumulation of sulphate sulphur, with the maximum residual concentration of 90 mg SO_4_^2−^-S/L being observed. The maximum ammonium nitrogen and nitrate nitrogen were 120 mg NH_4_^+^-N/L and 90 mg NO_3_^−^-N/L, respectively, with observed nitrification after 120 h resulting in residual ammonium concentration of 53 mg NH_4_^+^-N/L after 200 h. Denitrification of the nitrates was not observed, thus demonstrating incapacity of STK 03 to denitrify.

**Fig. 6:**
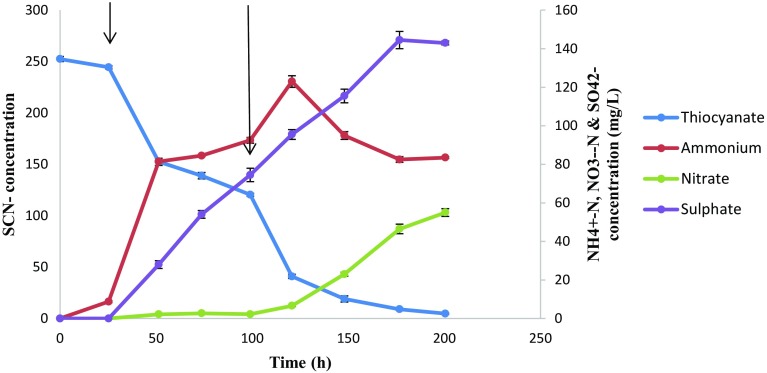
Thiocyanate degradation profile and formation of degradation products with the presence of free cyanide. *Error bars* represent deviations. The *arrows* represent cyanide spiking intervals

Thiocyanate degradation under the influence of free cyanide spiking was also evaluated (Fig. 6). Cyanide spiking was carried out at 25 and 100 h. Under these conditions, STK 03 had a degradation efficiency increase to 98 % from an initial concentration of 250 mg SCN^−^/L, meaning that the presence of free cyanide propagated thiocyanate degradation. It was hypothesised that this observation might be due to a metabolic shock response that might have triggered or upregulated the expression of thiocyanate degrading enzymes. The residual thiocyanate concentration was found to be 4.7 mg SCN^−^/L. Sulphates and nitrates accumulated throughout the experiments reached a maximum sulphate and nitrate concentration of 144.5 mg SO_4_^2−^-S/L and 55 mg NO_3_^−^-N/L. Thiocyanate degradation was accompanied by ammonium generation, resulting in a maximum ammonium concentration of 123 mg NH_4_^+^-N/L. Ammonium oxidation from 120 h was observed with a sudden increase in nitrates thereafter, although denitrification was not observed.

*Pseudomonas stutzeri* 18 and *putida* 21 were able to degrade SCN^−^ from an initial concentration of 60 mg SCN^−^/L with the terminal sulphur products from SCN^−^ degradation being thiosulfate and tetrathionate, respectively (Grigor’eva et al. 2006). In this study, the terminal sulphur product from SCN^−^ degradation was sulphates. This suggested that these organisms employ a different biochemical pathway for the degradation of SCN^−^.

## Conclusion

This study demonstrated the ability of *Pseudomonas aeruginosa* STK 03, which was originally isolated from an oil spill site contaminated with compounds containing cyano groups, and was able to degrade free cyanide and thiocyanate under alkaline conditions, achieving a BRE of 80 and 32 % from 250 and 450 mg CN^−^/L, respectively. Additionally, the SCN^−^ degradation efficiency was 78 and 98 % from non- and cyanide-spiked cultures, respectively. This was a first study on thiocyanate degradation under alkaline conditions by an organism belonging to the Pseudomonadaceae family. Additionally, STK 03 surpassed the stipulated free cyanide tolerance threshold of 200 mg CN^−^/L, making this organism valuable for application in large-scale wastewater treatment applications, particularly for wastewater containing free cyanide and thiocyanate. Furthermore, this study demonstrated that the presence of free cyanide accelerated thiocyanate degradation rates using the isolate under observation. This information is valuable in constituting microbial consortia for the degradation of cyanide containing wastewater. It is, however, recommended that: (1) simultaneous biodegradation of free cyanide, metal-complexed cyanide and thiocyanate in the same media be evaluated, (2) genes and enzymes involved in the biodegradation of free cyanide, ammonium oxidation and thiocyanate be investigated, (3) the degradation of cyanide and related compounds in continuous biofilm systems by the organism need to be evaluated, and (4) the mechanistic changes on thiocyanate degradation in the presence of free cyanide be further investigated at a genetic level.
